# Incidence and clinical relevance of heterotopic ossification after internal fixation of acetabular fractures: retrospective cohort and case control study

**DOI:** 10.1186/s13018-015-0202-z

**Published:** 2015-05-09

**Authors:** Dominik Baschera, Hooman Rad, Dermot Collopy, René Zellweger

**Affiliations:** Department of Orthopaedics and Trauma Surgery, Royal Perth Hospital, Perth, WA 6000 Australia; University of Western Australia, Crawley, WA 6009 Australia

**Keywords:** Acetabular fractures, Heterotopic ossification, Prophylaxis, Internal fixation, Trauma

## Abstract

**Objective:**

The aim of the study was to evaluate predictors and clinical relevance of heterotopic ossification (HO) in patients treated for acetabular fractures in a tertiary referral centre.

**Patients and methods:**

The study is a retrospective cohort study with a nested case–control study. All patients treated with internal fixation of acetabular fractures from January 2004 to October 2013. Ninety patients had postoperative imaging available at 6 and 12 months postoperatively and received no prophylaxis. Plain radiographs were used to grade HO. The Hip disability and Osteoarthritis Outcome Score (HOOS) was used to compare outcomes between patients suffering from HO with patients who did not.

**Results:**

Sixteen patients (17.7%) suffered from HO. According to the Brooker classification, 5 had class I, 4 class II, 3 class III and 4 class IV HO. Traumatic brain injury (TBI) was the only significant risk factor for developing HO (odds ratio (OR) 8.6, 95% confidence interval (CI) (1.693–43.753), *p* = 0.014). The HO rate in patients with an anterior (ilioinguinal) or posterior (Kocher-Langenbeck) surgical approach was 20% and 21% respectively, and the HO rate in patients with a combined approach was much lower at 11%. Neither fracture type nor gender nor age increased the risk of HO significantly. The outcome measured by HOOS was not significantly different between patients with HO and patients in the control group. Patients with HO Brooker class II–IV had slightly lower (effect estimate +4.25, 95% CI (−10.2 to +12.10), *p* = 0.220) HOOS compared to the majority of the control group.

**Conclusion:**

A very low rate of HO was found compared to the HO rates described in other studies with similar patient cohorts who received prophylaxis. Based on our findings and the current literature, we do not recommend giving prophylaxis against HO to patients after internal fixation of acetabular fractures.

## Background

Heterotopic ossification (HO) in patients receiving internal fixation of acetabular fractures is a potentially disabling complication that occurs in up to 58% of cases [1,2]. In many centres, indomethacin or low-dose radiotherapy is administered as prophylaxis to prevent the development of HO, although these practices are still controversial. Heterotopic ossification is a process by which the soft tissue becomes ossified. It occurs when primitive mesenchymal cells in the surrounding soft tissue are transformed into osteoblastic cells. Differentiation occurs within 16 h after surgery and peaks at 32 h [3]. The trigger for HO following major hip procedures is unknown, but several risk factors have been named. HO is commonly classified etiologically into neurogenic, musculoskeletal trauma related and hereditary. It is significantly more prevalent in patients who have sustained severe head traumas and spinal injuries. Lateral surgical approach, delay in internal fixation, diffuse idiopathic skeletal hyperostosis, Paget’s disease and hypertrophic osteoarthritis are other factors that may contribute to developing HO. A recent retrospective analysis found prolonged mechanical ventilation as a risk factor for developing HO [4]. The most common symptom of HO of the hip is stiffness of the joint with varying degrees of range of movement loss. Diagnosis after osteosynthesis is usually done radiographically with an anterior-posterior (AP) view of the pelvis and/or the hip. HO of the hip is classified using the Brooker classification grading HO from 1 to 4 according to the size of the bone islands in the soft tissue and their relative position to the acetabulum and the greater trochanter [5]. The only treatment option for established HO is delayed surgical resection when the heterotopic bone has matured and is encapsuled, which is approximately 6 months postoperatively [6].

In many centres, postoperative prophylaxis is administered. The type of medication and the duration of treatment recommended vary, but the most common prophylaxis is indomethacin in a 25-mg dose, three times daily (or slow release 75 mg once daily), for 7–14 days postoperatively, commenced within 24–48 h after the surgical procedure. While other non-steroidal anti-inflammatory drugs (NSAIDs) were found to be effective for the prevention of HO following total hip replacements, indomethacin remains the only drug with evidence in preventing HO following acetabular surgery [7]. A recent study compared the efficacy of indomethacin given for 3 days, 7 days and 6 weeks compared to placebo and found the best results in patients prescribed indomethacin for 7 days [8]. The worst results were found in patients that received indomethacin for 6 weeks. There was no reduction of symptomatic HO between groups that received indomethacin and placebo group [8]. In some centres, external beam radiation in a single dose of 700 to 800 centigray (cGy) is administered as prophylaxis for HO 24–72 h postoperatively. The current literature disputes the efficacy of different types of HO prophylaxis in patients with acetabular fractures receiving internal fixation.

However, the beneficial effect of HO prophylaxis after fixation of acetabular fractures has never been ascertained. Both types of prophylaxis have side effects such as gastric ulcers, renal toxicity and fracture non-union from NSAIDs and malignancy for irradiation, possible benefits need to be established [9-15]. The aim of our study was to assess the rate of HO in a population who did not receive a prophylaxis. We also aimed to compare risk factors and functional outcomes of patients receiving internal fixation for acetabular fractures who developed HO with patients who did not.

## Patients and methods

A retrospective study of patient notes and postoperative AP pelvic radiographs was performed for all patients documented on the theatre list for internal fixation of acetabular fractures over a 9-year period from January 2004 to October 2013. The study was approved by the Royal Perth Hospital review board. All procedures were performed in a single centre by a single surgical team. Patients that had follow-up imaging done in another hospital had to be excluded due to inaccessibility of necessary x-rays. The main inclusion criteria were the availability of at least two postoperative radiographs: one within 12 weeks and 6 months postoperatively during the typical period of HO development. Medical records were reviewed for risk factors of developing HO including head and spinal injuries, male sex, rheumatic diseases, interval from surgery to internal fixation and surgical approach. We also reviewed postoperative medications, in particular NSAIDs. Patients who received prophylaxis or NSAIDs postoperatively within the first 72 h, which is the usual period of HO induction, were excluded from the analysis. Patients who received NSAIDs later than 72 h postsurgery for more than once-off pain relief were also excluded.

**Figure 1:**
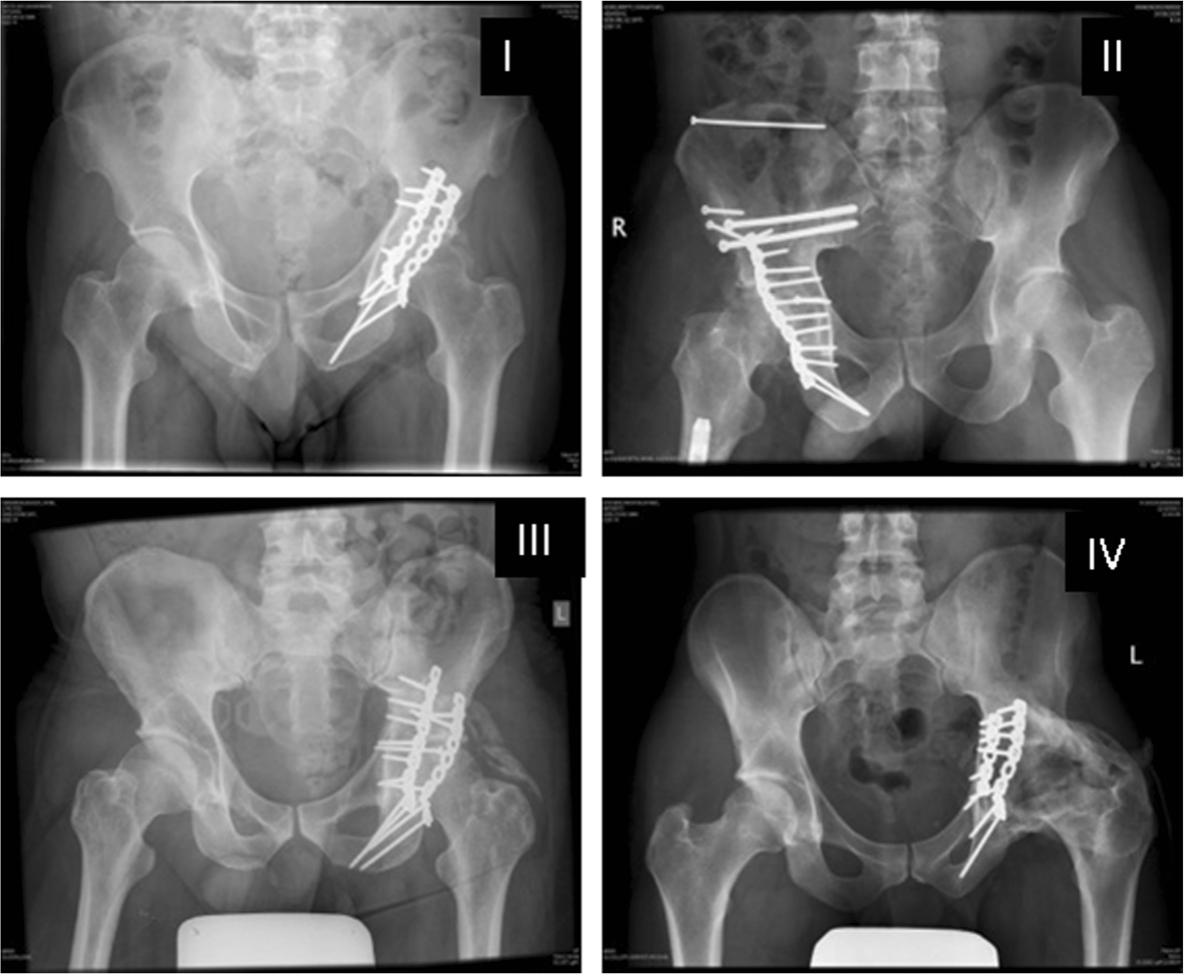
Different grades of heterotopic ossification according to the Brooker classification. I: X-ray of a Brooker class I heterotopic ossification (left hip): ‘islands of bone within soft tissues around the hip’. II: X-ray of a Brooker class II heterotopic ossification (right hip): ‘bone spurs in pelvis or proximal end of femur leaving at least 1 cm between the opposing bone surfaces’. III: X-ray of a Brooker class III heterotopic ossification (left hip): ‘bone spurs that extend from the pelvis or the proximal end of femur, which reduce the space between the opposing bone surfaces to less than 1 cm’. IV: X-ray of a Brooker class IV heterotopic ossification (left hip): ‘radiographic ankylosis of the hip’.

The Brooker classification was used to grade HO (Figure 1). The diagnosis was determined by postoperative plain AP view radiographs of the pelvis 1–12 months after the procedure. The definitive diagnosis was confirmed through a second opinion of an independent senior radiologist.

The patients’ symptoms and functional limitations were assessed and quantified using the Hip disability and Osteoarthritis Outcome Score (HOOS) [16,17]. The HOOS was used to assess the functionality of patients who developed HO and a randomly (using excel random function) selected group of a similar size (16 rounded up to 20) of the patients who did not.

The HOOS is a questionnaire survey intended to be used to assess the patient’s opinion about their hip and associated problems and to evaluate symptoms and functional limitations related to the hip [17]. The outcome score consists of 40 items assessing five subscales. The five separate patient-relevant dimensions are pain (P), symptoms (S), activity limitations daily living (ADL), function in sport and recreation (SP) and hip-related quality of life (QOL). Pain (P) includes 10 items with a total score of 40 points, symptoms (S) includes 5 items with a total score of 20, activity limitations of daily living (ADL) includes 17 items with a total score of 68 and finally function in sport and recreation (SP) and hip-related quality of life (QOL) both include 4 items with a total score of 16 each. Patients answered questions with the following options (0–4): no, mild, moderate, severe and extreme. To interpret the score, the outcome measure is transformed in a worst to best scale from 0 to 100, with 100 indicating no symptoms and 0 indicating extreme symptoms. To calculate the total HOOS score, the subscales need to be summed up, using the following formula for all dimensions.

$$ \mathbf{100} - \left[\left(\mathbf{patient}'\mathbf{s}\ \mathbf{s}\mathbf{core}\ \mathbf{of}\ \mathbf{the}\ \mathbf{s}\mathbf{ubscale} \times \mathbf{100}\right)/\left(\mathbf{total}\ \mathbf{s}\mathbf{core}\ \mathbf{of}\ \mathbf{the}\ \mathbf{s}\mathbf{ubscale}\right)\right] $$

The subscales can be plotted as a HOOS profile, by connecting the mean scores for all five dimensions with a line [17].

### Statistical analysis

All available data was analysed using SPSS_®_ (Version 21.0). For the descriptive analysis, categorical variables like Brooker classification, gender and surgical approach were compared to each other using Fisher’s exact test. To compare continuous factors like duration to internal fixation or age with the dichotomous outcome HO, we used the Mann–Whitney *U* test or Kruskal-Wallis test if there were more than two categories (e.g., surgical approach). The limit of significance was set to *p* ≤ 0.05.

## Results

**Figure 2:**
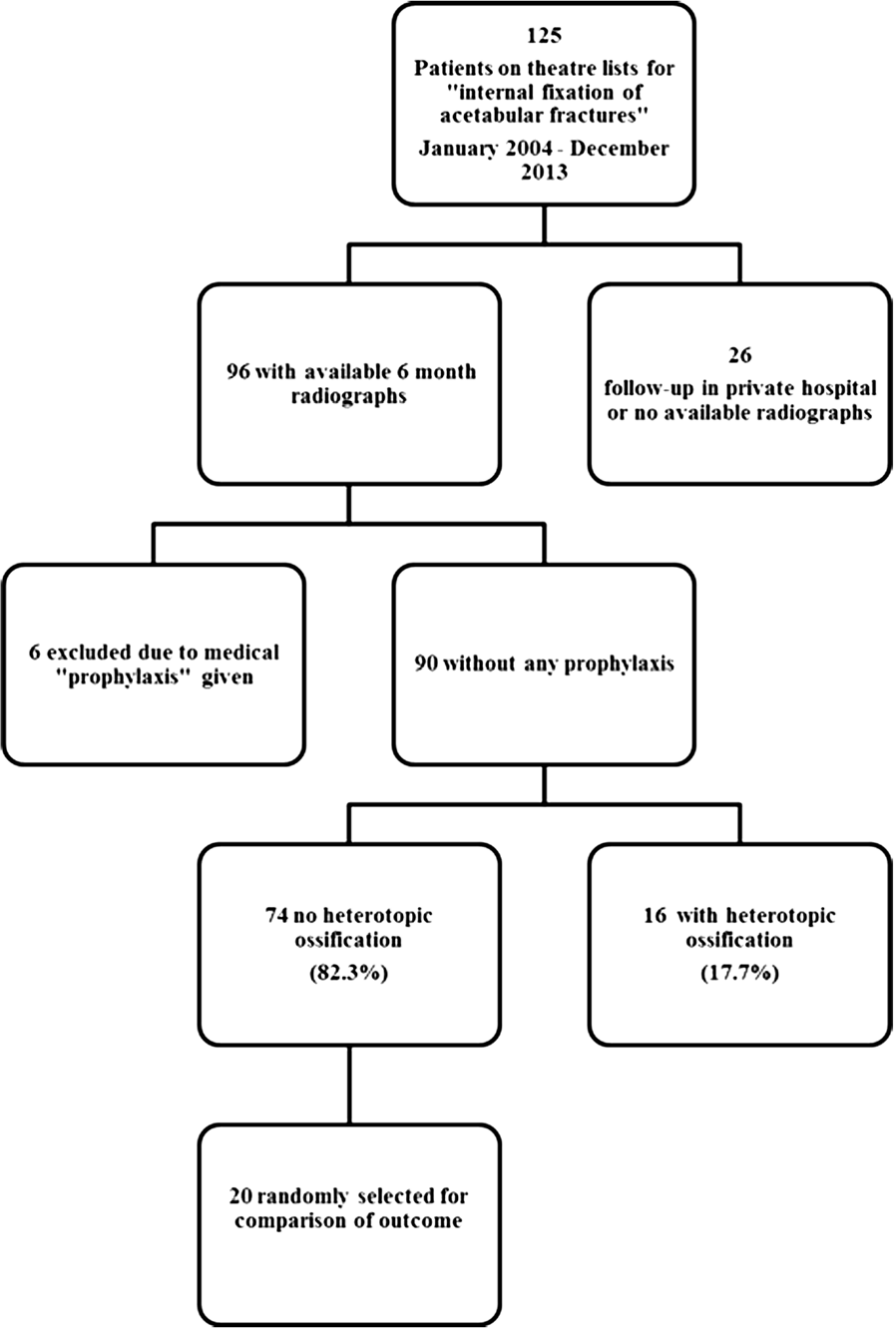
Flow chart displaying patient selection.

Patient selection is displayed in Figure 2. The mean age of the study population was 34.6 years (14–75). The male–female ratio was 3:1. The mechanism of injury was mainly road traffic related with 56 car and 28 motorbike accidents. The remaining patients sustained injuries in falls (5) or in a skateboard accident (1). The mean interval from injury to operation/internal fixation was 7.47 ± 4.38 days (range 0–19 days). Seven (7.1%) had also sustained concomitant traumatic brain injury (TBI) and 20 (22.2%) had sustained a spine trauma.

Heterotopic ossification was present in 16 patients (17.7%). According to the Brooker classification, 5 were classified as Brooker I, 4 as Brooker II, 3 as Brooker III and 4 as Brooker IV. There were no changes in grading 6 months after the trauma was sustained. The most common surgical approach was a posterior (Kocher-Langenbeck) approach (*n* = 58), followed by combined posterior-anterior approach (*n* = 27) and anterior (ilioinguinal) approach (*n* = 5). Similar rates of HO were found in anterior (20%) and posterior (21%) approaches, while patients with combined anterior-posterior approach, HO only developed in 11% of cases. In patients with and without HO, the fracture types were present in similar proportions, A2-type fractures (6/37.5% vs. 23/31.1%) being the most frequent followed by B2-type fractures (5/31.3% vs. 22/29.7%). None of the fracture types were associated with a higher risk of HO. Predictors of HO, their prevalence and unadjusted odds ratios in the groups with and without HO are displayed in Tables 1 and 2.

**Table 1:** **Different factors previously reported as risk factors for developing heterotopic ossification (HO)**

	**HO (** ***n*** **= 16)**	**No HO (** ***n*** **= 74)**	***p*** **value**	**95% CI**
Traumatic brain injury	4 (25%)	3 (4%)	*p* = 0.014*	
Spine injury	5 (31%)	15 (20.3%)	*p* = 0.337	
Male–female ratio	10:6 (69%♂; 31%♀)	56:18 (76%♂; 24%♀)	*p* = 0.351	
Interval injury to treatment (days mean ± SD)	9.188 ± 4.35	7.095 ± 4.33	*p* = 0.074; EE = −2	−4 to 0
Age (years mean ± SD)	39.875 ± 17.32	34.473 ± 13.30	*p* = 0.281; EE = −2	−13 to 4

*EE* effect estimate.

*Statistically significant; ♀ female; ♂male; Fisher exact test (two-sided).

**Table 2:** **Unadjusted odds ratios for different predictors of heterotopic ossification**

	**OR**	**95% confidence interval**		***p*** **value** ^**a**^
TBI	8.606	1.693	43.753	0.014*
Spine injury	1.788	0.539	5.933	0.258
Male–female	0.536	0.171	1.680	0.217
Combined approach	1.730	0.592	5.051	0.221
T-type fracture	0.951	0.425	2.132	0.561

^a^Fisher’s exact test (one-sided).

*Significant.

### Hip disability and Osteoarthritis Outcome Score (HOOS)

**Figure 3:**
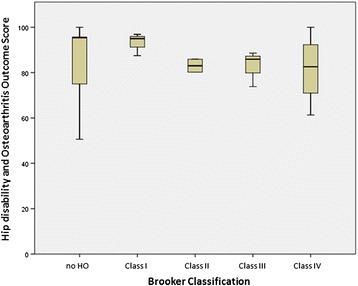
Hip Osteoarthritis Outcome Score according to the grade of heterotopic ossification (0 = none; 1–4 HO according to Brooker classification).

Response rate was 12/16 (75%) in the HO group and 13/20 (65%) in the randomly selected control group. There were no significant differences in age, male–female ratio and type of fracture in either group as well as between respondents and non-respondents. The HOOS in patients with HO and those in the control group were not statistically different (Table 3). Considering the grade of Brooker, patients suffering from Brooker grade II–IV HO had slightly lower HOOS (Figure 3). The characteristics and variables of the patients with HO are summarized in Table 4.

**Table 3:** **Hip disability and Osteoarthritis Outcome Score (HOOS)**

	**Mean**	**Median**	**±SD**	**Range**
HO	85.03	85.9	±10.52	61–100
Control	86.04	95.5	±15.33	51–100
*p* value	0.525	0.220		
Estimate		+4.25		
95% CI		−10.2 to +12.10

**Table 4:** **Characteristics and variables of patients with heterotopic ossification**

**Age**	**Sex**	**Mechanism**	**Interval to treatment (days)**	**Surgical approach**	**TBI**	**Spinal trauma**	**AO-OTA classification**	**HOOS**	**Brooker class**
42	f	MVA	10	Posterior	No	Spinal injury	A3	84.6	4
27	m	MVA	6	Posterior	No	Spinal injury	A2	95	1
16	f	MBA	9	Anterior	No	*Nil*	B2	87.5	1
45	m	MVA	4	Posterior	No	*Nil*	A2	x	2
62	m	MVA	4	Posterior	Yes	Spinal injury	A2	x	1
22	f	MVA	6	Posterior	No	*Nil*	A1	x	1
27	m	MBA	5	Posterior	No	*Nil*	A1	85.9	2
74	f	MVA	19	Posterior	No	*Nil*	A2	96.9^a^	1
47	f	MVA	7	Combined	No	*Nil*	B2	73.8	3
42	F	MVA	10	Combined	No	*Nil*	B2	85.9	3
35	m	MBA	10	Combined	Yes	Spinal injury	B2	x	2
67	m	MVA	12	Posterior	No	*Nil*	A2	80.6	4
52	m	MVA	13	Posterior	No	*Nil*	B2	88.6	3
35	m	MVA	16	Posterior	No	*Nil*	B1	x	2
21	m	MVA	5	Posterior	Yes	*Nil*	A2	61.3	4
24	m	MBA	11	Posterior	Yes	Spinal injury	A1	100	4

*MBA* motor bike accident, *MVA* motor vehicle accident, *TBI* traumatic brain injury.

^a^Patient had a total hip replacement 1 year after the accident; x = Hip and Osteoarthritis Outcome Score (HOOS) could not be obtained.

## Discussion

We found a relatively low HO rate of 17.7% (12.2% HO class ≥2) in patients with internally fixed acetabular fractures who received no prophylaxis. Concomitant TBI was significantly correlated with a higher risk of HO. The risk of HO in patients with combined anterior and posterior approach appeared to be lower than in patients with either anterior or posterior approach. Another trend observed was the slightly higher interval to treatment in the group of patients with HO. Surprisingly, the HOOS was not different between the HO and control group. Patients with HO graded II–IV according to the Brooker classification seemed to have slightly lower HOOS; however, this was not significant. Gender did not appear to influence occurrence of HO. Concomitant TBI was the strongest predictor found for HO.

Our overall rate of HO after internal fixation of acetabular fractures was low compared to the rates found in the literature [1,2]. It was reported to have an incidence as high as 90% in certain risk groups [18]. Karunakar et al., who summarized Brooker III-IV and excluded risk groups (TBI), found HO rates of 15.2%–19.4% (indomethacin vs. placebo) [19]. Another RCT compared HO in fracture patients treated with or without indomethacin prophylaxis had much higher HO rates of 47.4% and 56.8%, respectively [2]. In this study, CT volumetry was used and detected more clinically irrelevant HO class I patients. A meta-analysis including 13 articles classifying HO found an overall incidence of 25.6% [20]. A recent retrospective study in which none of the patients received prophylaxis found Brooker III–IV HO in 21% of patients with internal fixation of acetabular fractures [21]. Sagi et al. conducted a randomized controlled trial of patients treated with internal fixation of acetabular fractures. Patients were randomly placed into a placebo group or one of the three groups receiving indomethacin prophylaxis for the duration of 3 days, 1 week, and 6 weeks, respectively [8]. They reported rates of symptomatic HO (Brooker III–IV) in 19% of the placebo group and in 6%–31% of the three prophylactic groups and found no statistically significant reduction of symptomatic HO [8].

Patients with acetabular fractures and TBI had a much higher risk of HO. This has been observed in previous studies, and the causal correlation has at least partially been explained [22-25]. Other traditional risk factors like gender, higher age and T-type fracture reported in several studies seemed less meaningful [26]. The present study shows a lower HO rate among patients with a more extensive combined anterior and posterior approach which could mean that this approach might have been beneficial to these patients with regard to HO. This may be due to a more thorough debridement and more anatomical reduction in those patients. Similarly to a study by Daum et al., a longer surgical delay was also found in patients who developed HO [27]. Mears et al. already found that a surgical delay of more than 11 days resulted in fewer anatomical reductions [28]. Maybe a poor reduction rate in patients with longer surgical delay may have lead to a higher rate of HO.

The outcome (HOOS) in this study was not much different in patients with HO. Only patients with class III or IV HO had slightly lower scores. McLaren already found that HO grade I (Brooker classification) has no clinical relevance. He found no association of grade I HO with decreased range of motion of the hip or any loss of function. Grade II was associated with a loss of range of motion of the hip without loss of function whereas functional impairment was found in grade III and IV HO [29]. The latter findings were not replicated in the present study results, and therefore, the clinical significance of HO for the outcome in acetabular fracture patients seems questionable.

In the light of this, the possible side effects of any prophylaxis should be considered. In the case of radio-prophylaxis, one side effect is malignancy. Despite concluding that lifetime risk of radiation-induced cancer or infertility was insignificant, Oertel et al. found in a complex experimental model that prophylactic irradiation after fixation of an acetabular fracture in a 30-year-old patient results in a cumulative increased risk of solid cancer by 1% at the age of 65 [12]. Gastrointestinal and renal toxicity are known complications of treatment with NSAIDs [10,11,13,14]. In one study, compliance to medical prophylaxis was measured by control of indomethacin serum level and interviews. It was found that one third of the patients admittedly withdrew from taking indomethacin [19]. More than half of them did so because of side effects (13 compared to 1 in the placebo group). Two studies demonstrated that indomethacin prophylaxis significantly increases the risk of non-union in concomitant long-bone fractures, which are very common in acetabular fracture patients [9,15].

The strength of our study is the relatively large number of acetabular fractures reviewed in one centre and treated by one surgical team. This minimizes the risk of bias through individual differences or techniques of the surgical treatment. None of the patients included received indomethacin or irradiation prophylaxis. The limitations of our study are that it is non-randomized. Bias is possible due to the retrospective design and some loss to follow-up due to missing radiographs. Despite the relatively large number of patients with internally fixed acetabular fractures included, the number of patients with HO was low and therefore limiting the statistical power of our study. However, a study with the largest cohort which examined the effect of prophylaxis against HO in patients with internally fixed acetabular fractures failed to gain sufficient statistical power [19].

Instead of new research emphasizing on the kind of prophylaxis needed, randomized controlled studies should question if there is an effect of prophylaxis at all. Possible risk factors like surgical approach and surgeon’s skills (invasiveness, thoroughness of debridement) could explain higher HO rates found in earlier studies. The influence of the time interval from injury to treatment should be considered, and early fixation without unnecessary delay should be aimed for. From our results, we cannot recommend giving prophylaxis against HO to patients after internal fixation of acetabular fractures. The question of whether prophylaxis has any beneficial effect for patients considered high risk for developing postoperative HO, such as patients with TBI, is not yet proven.

## Conclusion

A low rate of HO was found in this patient cohort that received no prophylaxis in comparison to HO rates described in other studies, which included similar patient groups that received prophylaxis. Traumatic brain injury was the only confirmed factor that increased the risk of HO significantly. Longer intervals from injury to internal fixation were observed in patients with HO. An important measure to prevent HO might be to avoid unnecessary delay of internal fixation of the fractures. Based on our findings and the current literature, giving patients routine prophylaxis against HO after internal fixation of acetabular fractures is not recommended.

## Abbreviations

HO: Heterotopic ossification
HOOS: Hip disability and Osteoarthritis Outcome Score
AP: Anterior-posterior
cGy: Centigray
NSAIDs: Non-steroidal anti-inflammatory drugs
